# Social Progress Index as a Determinant of Healthcare Access and Treatment in Pancreatic Cancer

**DOI:** 10.3390/curroncol33060346

**Published:** 2026-06-09

**Authors:** Francisco Tustumi, Felipe Antonio Boff Maegawa, Victória Bulcão Caraciolo, Giovanna Mennitti Shimoda, Isabella Paes Leme Rufino, Bianca Aguiar Giacometti dos Santos, Lucas Cata Preta Stolzemburg, Daniel José Szor, Sergio Eduardo Alonso Araujo, Pedro Luiz Serrano Uson Junior, Nelson Wolosker

**Affiliations:** 1 Department of Health Sciences, Einstein Hospital Israelita, São Paulo 05652-900, SP, Brazilisabella.prufino@einstein.edu.br (I.P.L.R.); bianca.gsantos@einstein.edu.br (B.A.G.d.S.);; 2 Department of Surgery, Emory University, Atlanta, GA 30322, USA

**Keywords:** social determinants of health, health disparities, health inequities, socioeconomic status, pancreatic neoplasms

## Abstract

**Background**: Health accessibility is a key determinant of equitable cancer care. In many countries, specialized oncology services are concentrated in urban and socioeconomically advantaged regions, forcing many patients to travel long distances for treatment. Consequently, geographic and social characteristics may be impactful in determining cancer healthcare outcomes. **Objective**: The aim of this study was to evaluate the association between the municipal-level Social Progress Index (SPI) and geographic travel burden, stage at diagnosis, treatment, and survival in patients with pancreatic cancer in São Paulo state, Brazil. **Methods**: We conducted a population-based study using data from “Fundação Oncocentro” on adults with pancreatic adenocarcinoma (2005–2025). The SPI (0–100 scale), a composite measure of municipal social and environmental development, was the primary exposure. It is structured into 3 dimensions and 12 components: Basic Human Needs (nutrition, medical care, water and sanitation, housing, safety); Foundations of Well-being (education, information access, health, environmental quality); and Opportunity (rights, freedom of choice, social inclusion, higher education). Municipal residence and cancer center locations were geocoded, and travel distance (km) was estimated. Multivariable Cox, logistic, and linear regression models assessed associations between SPI and overall survival, stage IV at diagnosis, surgery, and travel distance. **Results**: A total of 13,478 patients were included (mean follow-up 15.1 ± 27.2 months; mean age 62.3 years; 50.4% male). Stage IV disease was frequent (46.3%), and surgery was performed in 33% of cases. Over half of patients (53.2%) traveled more than 10 km for treatment. Increasing SPI was strongly associated with shorter travel distance (β −62.6 km per SPI unit; *p* < 0.001) and higher odds of surgery (OR 1.04; *p* < 0.001) and remained independently associated with a higher likelihood of undergoing surgical treatment (adjusted OR 1.04; *p* < 0.001). The proportion of stage IV disease did not decrease with increasing SPI and was slightly higher in the highest quartile (49.3%). In survival analysis, SPI demonstrated a protective effect in univariate modeling (HR 0.987; *p* < 0.001), but lost significance in multivariable analysis (*p* = 0.125). Travel burden was not retained as an independent predictor of survival after adjustment. **Conclusions**: Municipal-level SPI was a strong determinant of healthcare access and the likelihood of receiving surgical treatment for pancreatic cancer. Social and geographic vulnerability directly influence care pathways, revealing structural inequities in access to treatment. SPI-based stratification may serve as a practical tool to identify priority regions for transport support and equitable allocation of oncology services.

## Simple Summary

Pancreatic cancer is one of the most serious cancers, and the best chance of cure usually depends on complex surgery offered in only a few specialized hospitals. In a country as large as Brazil, many patients live far from these centers, often in towns with fewer resources. We studied more than thirteen thousand people with pancreatic cancer in São Paulo state and measured the social development of the town where each person lived. People from less developed towns traveled much farther for care and were less likely to undergo surgery, even though the public health system is free for everyone. How advanced the cancer was and how long patients survived were driven mostly by the aggressive nature of the disease. Our findings show that a simple measure of a town’s social development can help identify the communities that most need transport support and better organized cancer services.

## 1. Introduction

Timely and effective access to oncologic care is a fundamental component of health equity [1]. However, in large and socioeconomically diverse countries such as Brazil, geographic barriers remain a major obstacle to equitable cancer treatment. Specialized oncology centers are unevenly distributed, often concentrated in wealthier and more urbanized regions, requiring many patients to travel long distances for diagnosis, treatment, and follow-up [2]. Barriers to healthcare access may lead to delays in care, treatment interruptions, financial hardship, and ultimately poorer outcomes, particularly among vulnerable populations [3].

Brazil’s health system is mixed. The publicly funded Unified Health System (Sistema Unico de Saude, SUS) provides universal, tax-financed care that is free at the point of use and is the sole source of coverage for roughly three quarters of the population. A parallel private supplementary sector, financed by private health insurance and out-of-pocket payments, covers approximately one-quarter of Brazilians and is concentrated in wealthier, more urbanized regions. High-complexity oncologic care—including pancreatic cancer surgery—is delivered predominantly through the SUS and is regionally centralized; consequently, geographic and socioeconomic barriers fall disproportionately on patients who depend exclusively on the public system. Although Brazil’s Unified Health System has made substantial progress in expanding access to oncology services, systematic assessments of spatial disparities and patient travel burden remain scarce and methodologically heterogeneous—existing studies vary in scope and geographic coverage, are frequently restricted to selected regions or tumor types, and rarely incorporate municipal-level socioeconomic context [4,5]. Existing health system indicators typically focus on service availability but fail to capture patients’ lived experience in accessing care within their geographic context. Furthermore, municipal-level socioeconomic and healthcare infrastructure factors are rarely integrated into national analyses of access to oncologic care [6].

In this context, predictive models can integrate complex, multilevel data to estimate social progress and identify municipalities facing disproportionately high geographic barriers to cancer care. Such tools can inform targeted interventions—such as transportation subsidies for specific regions and patient populations—and support evidence-based planning and decision-making.

Healthcare barriers to oncologic care constitute a pervasive yet underexplored determinant of global health inequities, particularly in low- and middle-income countries (LMICs) with continental-scale territories. In Brazil—a country spanning 8.5 million km^2^ and characterized by fragmented transportation infrastructure—patients with cancer diagnoses face disproportionate access to specialized centers, which are typically concentrated in urban hubs.

Within this framework, pancreatic cancer is a particularly informative model for studying barriers to access. Pancreatic cancer is among the most lethal solid tumors, and its management is time-sensitive and resource-intensive, depending on complex, centralized, high-volume surgical and multidisciplinary care [7]. In addition, curative-intent treatment is concentrated in a small number of specialized high-technology centers, and consequently, pancreatic cancer potentially behaves as a ‘tracer’ condition that magnifies the impact of geographic and socioeconomic barriers along the care pathway. Pancreatic cancer is a highly aggressive malignancy, with adenocarcinoma accounting for approximately 85% of diagnosed cases, affecting the head predominantly, but also the body and tail of the pancreas [8]. Survival rates remain low, as approximately 80% of cases are unresectable at diagnosis, and even among those undergoing curative-intent resection, only 27% achieve five-year survival [9]. Additionally, cancer incidence, diagnosis, and outcomes vary significantly across racial and ethnic groups, reflecting disparities in socioeconomic conditions, healthcare access, exposure to risk factors, and barriers to prevention, early detection, and treatment [10].

In Brazil, it is estimated that 3.1% of the municipalities have high-complexity oncology services, while the remainder lack the capacity to provide oncologic treatments, compelling rural and socioeconomically disadvantaged populations to undertake multi-day journeys to access treatment. These systemic barriers contribute to advanced-stage diagnoses, high treatment abandonment rates, and high mortality rates [11].

This study addresses a critical gap in health policy: although some LMICs, such as Brazil, have expanded theoretical access to oncologic care under universal health systems, the actual geographic accessibility of these services remains poorly quantified. Existing metrics fail to account for the interaction between transportation inefficiencies, socioeconomic deprivation, and clinical outcomes. This study aims to evaluate the association between the municipal-level Social Progress Index (SPI) and geographic travel burden, stage at diagnosis, and treatment in patients with pancreatic cancer in São Paulo state, Brazil.

## 2. Methods

### 2.1. Study Design and Setting

We conducted a population-based retrospective study using data from the Fundação Oncocentro de São Paulo (FOSP), which compiles information from cancer centers across the State of São Paulo, Brazil. The study included patients diagnosed with pancreatic cancer between January 2005 and January 2025. São Paulo is the most populous and economically developed state in Brazil and serves as a major referral hub for oncologic care, receiving patients from both within and outside the state.

### 2.2. Research Question and Hypothesis

We examined whether municipal-level social progress, measured by the SPI, is associated with the pancreatic cancer care pathway—specifically, travel burden, stage at diagnosis, receipt of surgical treatment, and overall survival. We hypothesized that social progress shapes access beyond mere geographic distance to specialized centers: that is, that the SPI captures structural determinants (healthcare infrastructure, education, connectivity, and economic resources) influencing whether patients can reach and complete complex oncologic care. Accordingly, the primary objective was to identify and quantify these structural inequities to inform the prioritization of regions and populations for targeted support, rather than to estimate clinical effectiveness or the number of deaths that might be avoided.

### 2.3. Eligibility Criteria

We included adult patients with a confirmed diagnosis of pancreatic adenocarcinoma registered in the FOSP database. Cases were identified based on tumor site and histological classification. Patients with non-adenocarcinoma histologies or missing key variables (municipality of residence or treatment center) were excluded.

### 2.4. Data Sources

Clinical and demographic data were obtained from FOSP and included age, sex, tumor stage at diagnosis, histological subtype, and treatment modalities (surgery, chemotherapy, radiotherapy, and immunotherapy).

Municipal-level socioeconomic characteristics were assessed using the Social Progress Index (SPI), a composite measure of social and environmental development based on the methodology of the Social Progress Imperative. The Social Progress Imperative is a United States-based nonprofit organization, founded in 2012, that designs and disseminates the SPI framework. The SPI was developed to measure social progress beyond economic indicators, focusing exclusively on outcome-based social and environmental data. The global framework has been disseminated since 2013 in collaboration with Foundation Avina, Massachusetts Institute of Technology, and Harvard Business School, and is currently applied to more than 170 countries. Beyond national rankings, the framework has been adapted across multiple domains and geographic scales—including subnational and regional indices (such as the European Commission’s EU Regional Social Progress Index), municipal indices (such as the Brazilian Indice de Progresso Social, IPS), corporate social-impact and sustainability assessments, and as a complementary tool for monitoring the United Nations Sustainable Development Goals. In Brazil, the SPI was adapted to the national context to assess all 5570 municipalities using publicly available data. The 2025 Brazilian SPI is composed of 57 indicators aggregated into an overall score ranging from 0 to 100, structured across three dimensions—Basic Human Needs, Foundations of Well-being, and Opportunity—and 12 components, including nutrition and basic medical care, water and sanitation, housing, personal safety, access to basic knowledge, access to information and communication, health and wellness, environmental quality, individual rights, personal freedom and choice, social inclusion, and access to higher education (see Table 1). In 2025, Brazil achieved a score of 72.35 in the global SPI, ranking 55th among 170 countries.

Patients were stratified according to quartiles of the SPI to evaluate gradients in clinical characteristics, treatment patterns, and travel burden. SPI quartiles were defined based on the distribution of SPI values in the study population and categorized as Q1 (lowest), Q2, Q3, and Q4 (highest). Comparisons across quartiles were used to explore socioeconomic disparities in access to care and outcomes.

As shown in Table 1, the SPI and its three dimensions and twelve components constitute only one of several domains of municipal-level information compiled for this study; the remaining domains (e.g., healthcare infrastructure, connectivity, and environment) were assembled to characterize the municipalities and to support exploratory analyses, while the composite SPI served as the primary exposure in all regression models. Some indicators are conceptually related across domains—for example, ‘Water and Sanitation’ within the Basic Human Needs dimension of the SPI overlaps with ‘Adequate Sanitation’ listed under Housing and Infrastructure. This reflects the structure of the underlying IPS methodology, in which each SPI component is a normalized composite aggregated from multiple raw source indicators, whereas the items listed under the descriptive domains are the individual source variables. To avoid redundancy and collinearity, only the composite SPI—not its overlapping source indicators—was entered into the multivariable models.

### 2.5. Geocoding and Travel Distance Estimation

Municipality of residence and treatment facility were used to define origin–destination pairs. The locations were defined at the municipality level, using the centroid of the municipality of residence. The primary metric used in the analysis was the straight-line (Euclidean) distance between origin and destination, expressed in kilometers.

### 2.6. Outcomes

Geographic distance (km) from the patient’s residence to the treating cancer center was evaluated as a measure of access to care. Travel burden was further categorized into four predefined groups: ≤10 km, 10–50 km, 50–100 km, and >100 km.

Overall survival was defined as the time from the date of diagnosis to death from any cause. Follow-up time was calculated from the date of diagnosis to the date of last follow-up, and expressed in months. Patients who were alive at the time of last follow-up were censored. Vital status at last follow-up was also used to classify patients as alive or deceased for descriptive purposes only.

Metastasis (stage IV) at diagnosis was analyzed as a binary outcome.

Receipt of surgical treatment was used as a proxy marker of access to curative-intent therapy and was defined as the performance of any oncologic resection during care.

### 2.7. Statistical Analysis

Continuous variables are presented as mean (standard deviation), and categorical variables as counts and percentages. Comparisons across SPI quartiles were performed using analysis of variance (ANOVA) for continuous variables and chi-square tests for categorical variables.

Survival probabilities were estimated using the Kaplan–Meier method and compared using the log-rank test. Multivariable Cox proportional hazards models were used to evaluate factors independently associated with overall survival.

Logistic regression models were used to assess factors associated with stage IV disease at diagnosis and receipt of surgical treatment. Linear regression models were used to evaluate factors associated with travel distance (km).

SPI was analyzed both as a continuous variable and stratified into quartiles. Variables included in multivariable models were selected based on statistical significance in univariate analyses (*p* < 0.05).

Missing data were handled using a complete-case approach. Variables with missing values were not imputed, and analyses were performed using all available observations for each specific model. For time-to-event analyses, only patients with available dates of diagnosis and last follow-up were included to ensure accurate calculation of survival time. All analyses were conducted using R (version 4.3.3).

### 2.8. Ethics and Data Governance

This study used de-identified data from a public registry. According to Brazilian regulations, studies using publicly available anonymized data do not require formal ethical approval or informed consent.

## 3. Results

### 3.1. Population Characteristics

The study included 13,478 patients with pancreatic cancer treated within the FOSP network, with a mean age of 62.3 years (±12.9) and a balanced sex distribution (50.4% male). The mean SPI was 66.4 (±2.9). A high proportion of patients presented with advanced disease, with 46.3% diagnosed at stage IV, whereas only 7.6% were diagnosed at stage I. Surgical treatment was performed in 33% of cases, and nearly half of the patients received chemotherapy (48.3%). Regarding travel burden, more than half of the patients (53.2%) traveled distances greater than 10 km to receive treatment, and 24.4% experienced extreme travel burden (>100 km). Table 2 summarizes the baseline characteristics of the cohort.

### 3.2. Geographic Treatment Flows

**Figure 1:**
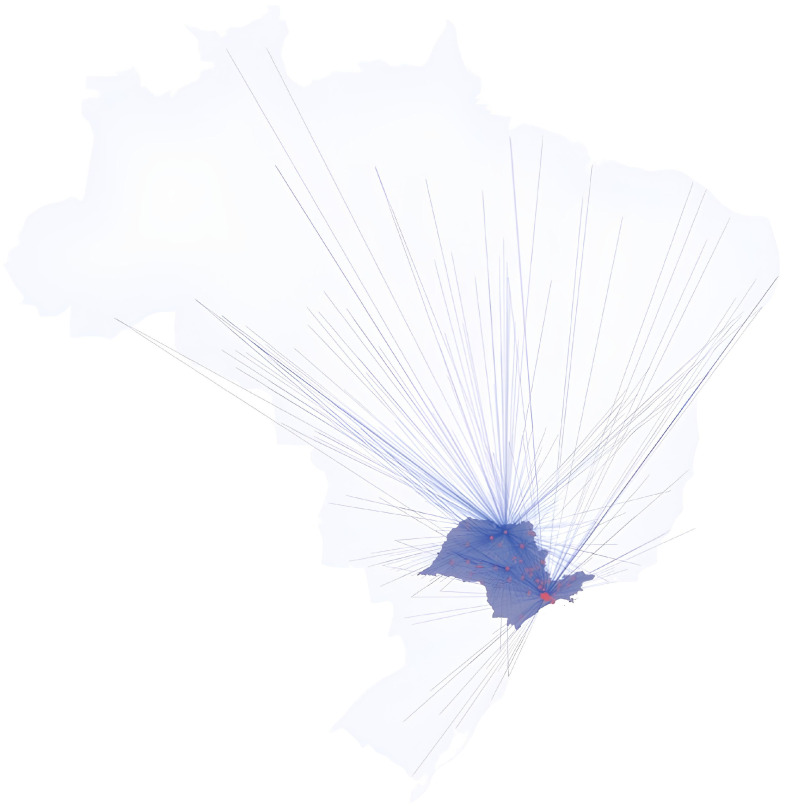
Origin–destination travel flows for pancreatic cancer treatment in the FOSP network, São Paulo State, Brazil. Each line represents the path between the municipality of residence and the attending cancer center. Line thickness is proportional to flow intensity. Destination nodes (treatment centers) are shown as semi-transparent circles; darker areas represent a higher volume of referrals. The state of São Paulo is highlighted in contrast to other Brazilian states. (No copyright issues.)

Figure 1 illustrates the origin–destination flows for pancreatic cancer treatment within the FOSP network. A strong concentration of treatment centers is observed in larger municipalities.

### 3.3. Association Between SPI and Clinical and Geographic Outcomes

**Figure 2:**
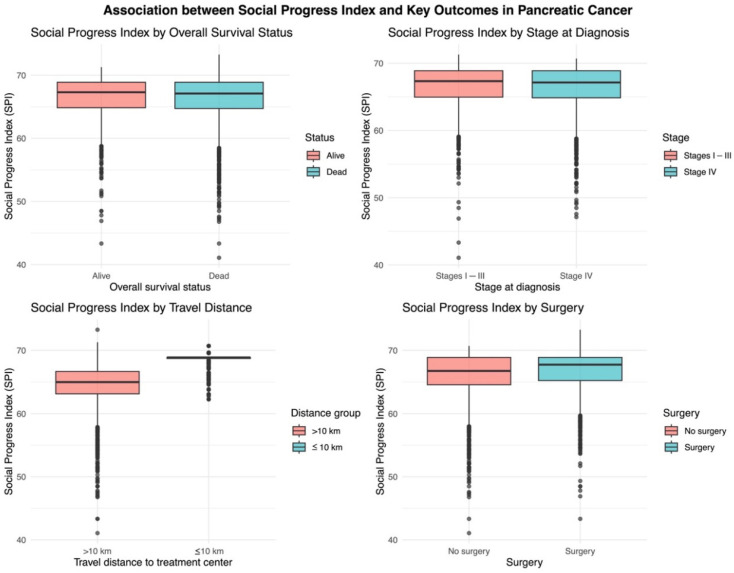
Distribution of the Social Progress Index (SPI) across key clinical and geographic outcomes in pancreatic cancer. The (**top**–**left**) panel shows SPI values by survival status (alive vs. deceased), while the (**top**–**right**) panel shows SPI by stage at diagnosis (Stages I–III vs. Stage IV). The (**bottom**–**left**) panel presents SPI according to travel distance to the treating center (≤10 km vs. >10 km), and the (**bottom**–**right**) panel illustrates SPI distribution by receipt of surgical treatment (surgery vs. no surgery). Each boxplot displays the median, interquartile range, and outliers as individual points. Higher SPI values represent better municipal–level social development. The figure explores potential associations among social progress, disease severity, access to treatment, and therapeutic patterns. (No copyright issues.)

Figure 2 presents the distribution of the SPI according to clinical and access-related outcomes. Patients who were alive exhibited, on average, higher SPI values compared to those who died. Similarly, individuals diagnosed at stages I–III had higher SPI scores than those diagnosed at stage IV. A clear gradient in SPI was also observed according to travel burden: patients who traveled ≤10 km for treatment had significantly higher SPI values than those who traveled longer distances. Finally, patients who underwent surgical treatment showed an SPI distribution shifted toward higher values compared to those who did not undergo surgery, suggesting an association between more favorable municipal socioeconomic conditions and increased access to surgical care. These comparisons are descriptive, illustrating the distribution of SPI values across outcome groups; formal statistical inference, including multivariable adjustment, is presented in the regression analyses (Table 3).

### 3.4. Analysis by SPI Quartiles

Stratification of the sample by SPI quartiles revealed a consistent socioeconomic gradient (Table 4). Patients in the lowest quartile (Q1 ≤ 64.7) were slightly younger and predominantly male. Although the proportion of stage IV disease was high across all quartiles, no clear inverse gradient was observed. Access to surgical treatment varied substantially across quartiles, with only 27.9% of patients in Q1 undergoing surgery compared to 35.6% in Q4 (*p* < 0.001). A similar pattern was observed for chemotherapy, with higher utilization in intermediate and upper quartiles. The most pronounced differences were observed in travel burden. Patients in Q1 traveled a mean distance of 359.4 km for treatment, whereas those in Q4 traveled only 24.8 km (*p* < 0.001). More than half of patients in the lowest quartile experienced extreme travel burden (>100 km), compared to only 4.5% in the highest quartile, highlighting marked geographic inequality associated with municipal socioeconomic conditions. As expected, lower travel burden was concentrated in the higher SPI quartiles, which correspond to larger and more urbanized municipalities, whereas patients in the lowest quartile—typically residing in smaller, less urbanized municipalities—travelled markedly farther (mean 359.4 km in Q1 vs. 24.8 km in Q4).

### 3.5. Overall Survival

In univariate analysis of overall survival, older age, female sex, stage IV disease, and absence of surgical treatment were associated with increased mortality risk. Therapeutic interventions—including surgery, chemotherapy, radiotherapy, and immunotherapy—were associated with significant protective effects. Continuous SPI was inversely associated with mortality (HR 0.987; *p* < 0.001), indicating a lower risk of death in municipalities with higher social progress. However, in the multivariable model, SPI was no longer statistically significant (HR 0.994; *p* = 0.125). See Table 3.

### 3.6. Stage IV at Diagnosis

In the analysis of factors associated with stage IV diagnosis, older age, female sex, and absence of treatment were associated with a higher likelihood of advanced disease. SPI showed a protective trend in univariate analysis (OR 0.987; *p* = 0.068) but did not remain independently associated after adjustment. Distance traveled to the treatment center was also not independently associated with advanced-stage diagnosis. See Table 3.

### 3.7. Determinants of Travel Distance and Surgical Treatment

SPI demonstrated a strong association with travel distance. Each one-unit increase in SPI was associated with an average reduction of approximately 62.6 km in travel distance (*p* < 0.001). In the analysis of factors associated with surgical treatment, SPI remained a strong independent predictor (OR 1.040; *p* < 0.001), even after adjustment for age, sex, disease stage, and other treatment modalities. Stage IV disease was the strongest negative predictor of surgery, while younger age and chemotherapy were also associated with a higher likelihood of undergoing surgical intervention. See Table 3.

## 4. Discussion

In this population-based study of 13,478 patients with pancreatic cancer treated within the FOSP network, municipal socioeconomic context, captured by the SPI, emerged as a strong determinant of structural access to pancreatic cancer care. Lower SPI was consistently associated with substantially greater travel burden and a lower likelihood of surgical treatment, indicating that patients from more vulnerable municipalities face important barriers in reaching specialized oncologic services. These findings highlight that, even within a universal health system, geographic and social inequalities remain deeply impactful for cancer care and directly influence access to potentially curative treatment.

The association observed between the SPI and both travel burden and access to surgical treatment likely reflects the combined effect of multiple structural domains embedded within the index. Elements related to healthcare infrastructure are particularly relevant: municipalities with a higher density of hospitals, physicians, oncologists, and surgeons are more likely to provide local or regional access to diagnostic and therapeutic services, thereby reducing the need for long-distance travel. Conversely, in areas with limited healthcare capacity, patients are more frequently referred to distant tertiary centers, increasing travel burden and potentially introducing delays or barriers in the care pathway. This mechanism is consistent with our findings showing a strong inverse association between SPI and travel distance, suggesting that geographic disparities in service distribution are a central driver of access inequalities.

In addition to infrastructure, domains related to education, connectivity, and socioeconomic conditions may influence the patient’s ability to navigate the healthcare system and reach specialized care. Lower levels of education, higher dropout rates, and reduced access to information and communication technologies may limit health literacy, delay symptom recognition, and impair the ability to engage effectively with referral systems [12,13]. Similarly, economic constraints reflected by lower GDP per capita and weaker labor market indicators may restrict access to transportation, lodging, and other indirect costs associated with seeking care in distant centers [14,15]. These barriers are particularly relevant in complex oncologic conditions such as pancreatic cancer, where timely referral to specialized surgical teams is essential. As a result, patients from lower-IPS municipalities may face cumulative disadvantages that reduce the likelihood of reaching centers capable of performing high-complexity procedures. Prior studies have examined these mechanisms directly: socioeconomic position, education, and insurance status have been linked to the likelihood of reaching high-volume centers and receiving resection [10,16], and Australian population-based data identified socioeconomic disadvantage and remoteness as determinants of whether patients with non-metastatic pancreatic cancer underwent attempted resection [17].

Weaker social support structures and reduced access to community resources may limit the patient’s capacity to adhere to complex treatment pathways, including surgical care that requires preoperative optimization and postoperative follow-up. Taken together, these findings suggest that SPI captures not only economic development but also the multidimensional context that determines whether patients can realistically access specialized oncologic care. The observed association between higher SPI and increased likelihood of surgery therefore likely reflects a combination of improved geographic access, greater system navigation capacity, and fewer structural barriers to completing complex treatment pathways.

The lower likelihood of surgical treatment observed among patients from municipalities with lower SPI is consistent with robust evidence demonstrating that social determinants of health directly influence the access to high-complexity oncologic care [18]. In a large National Cancer Database analysis, socioeconomic factors influenced not only whether patients underwent pancreatectomy but also where they received care, with patients treated at academic centers traveling significantly longer distances (mean 80.9 vs. 31.7 miles, *p* < 0.0001) and achieving better perioperative and survival outcomes [16]. Importantly, vulnerable populations—including Hispanic patients and those with greater comorbidity burden—were less likely to reach these specialized centers, highlighting how social and clinical factors jointly determine access to optimal surgical care [19]. These findings align with broader evidence indicating that social determinants such as income, education, insurance status, and race influence multiple steps of the pancreatic cancer care continuum, including access to high-volume centers, likelihood of receiving surgical resection, and delivery of guideline-concordant therapy [10]. In this context, our results reinforce that geographic distance is not an isolated barrier but rather a manifestation of underlying social and structural inequities. The observed association between lower SPI, greater travel burden, and reduced likelihood of surgery likely reflects the cumulative effect of these determinants, which limit patients’ ability to access high-quality care and, consequently, potentially curative treatment.

Our findings are consistent with international evidence on geographic and socioeconomic disparities in pancreatic cancer care. In the United States, Makar et al. showed that socioeconomic factors influenced both whether and where patients underwent pancreatectomy, with care at academic centers requiring substantially longer travel [16]. In Australia—another large country with a universal health system—Burmeister et al. found, in population-based analyses, that socioeconomic disadvantage and greater remoteness were associated with lower-quality care and a lower likelihood of attempted resection, as well as poorer survival, among patients with pancreatic cancer [17,20]. These parallels, across health systems as different as those of the United States, Australia, and Brazil, suggest that the structural disadvantage captured by composite social indices is a generalizable barrier to high-complexity cancer surgery rather than an artifact of any single financing model. Our study extends this literature by using a multidimensional, municipality-level social progress index—rather than income or insurance status alone—and by jointly modeling travel burden, stage, surgery, and survival within a single large population-based cohort.

Despite these marked disparities in access, neither SPI nor travel burden were independently associated with stage at diagnosis or overall survival after adjustment. This apparent dissociation is likely explained by the biological behavior of pancreatic adenocarcinoma, in which early-stage disease is uncommon, and symptoms typically arise at advanced stages regardless of geographic or socioeconomic context [21]. In our cohort, nearly half of patients presented with stage IV disease, and the analysis by SPI quartiles did not demonstrate a consistent gradient in stage IV disease across socioeconomic strata, reinforcing that tumor biology remains the dominant determinant of prognosis and may attenuate the measurable impact of access-related variables on survival outcomes. For less aggressive types of cancer, the social variables can be more impactful in predicting stage at diagnosis. Scoggins et al. [22], in a retrospective study, evaluated patients with breast, colorectal, and lung cancer, and found that >1 h driving or >100 miles were associated with advanced disease at diagnosis. Stitzenberg et al. [23] found that a 1-mile increase in distance from the cancer treatment location was associated with a Breslow thickness increase by 0.6% in melanoma patients. Taken together, these findings suggest that any survival-related benefit associated with higher social progress operates primarily through improved access to treatment—particularly the receipt of curative-intent surgery—rather than through earlier diagnosis, since stage at diagnosis showed no consistent gradient across SPI quartiles. Because the registry does not capture treatment timeliness, completion, or follow-up visits, therapeutic adherence could not be measured directly; nevertheless, the strong and independent association between SPI and receipt of surgery is most consistent with access to and engagement with the treatment pathway, rather than earlier detection, being the dominant mechanism.

Following the same rationale, the lack of an independent association between travel burden and survival in our study likely reflects the unique biological behavior of pancreatic adenocarcinoma, in which late-stage presentation is common regardless of geographic access, thereby attenuating the measurable impact of distance on survival outcomes. In contrast, for other malignancies, geographic access has been shown to directly influence prognosis. Baade et al. [24] demonstrated a 6% increase in mortality risk for each additional 100 km from the nearest radiotherapy facility in rectal cancer, while Lee et al. [25] showed worse overall survival among patients with lymphoma living in smaller or less accessible regions. More broadly, the implications of our results are likely to vary by tumor biology. For cancers that are more often detected early and have effective stage-dependent treatments—such as breast, colorectal, melanoma, and some lymphomas—social and geographic barriers may exert a larger measurable effect on stage at diagnosis and survival, as reported in the studies cited above [22,23,24,25]. In pancreatic cancer, by contrast, the dominant influence of aggressive tumor biology compresses these survival differences, even though the impact on access to treatment and on patient experience remains substantial. The SPI-based access framework proposed here is therefore generalizable across tumor types, but its survival implications should be interpreted in light of each disease’s natural history.

The absence of an independent survival effect in our study should not be interpreted as evidence that travel burden is clinically irrelevant. The relevance of travel burden extends beyond its direct association with stage at diagnosis or overall survival. Even in scenarios where distance does not independently affect survival, long travel distances impose a substantial clinical, psychological, and logistical burden on patients. In a recent systematic review including 23 studies on radiation therapy, patients were found to travel a median distance of approximately 20 miles, and greater travel distance was associated with reduced adherence to recommended treatment in multiple studies, with five studies demonstrating worse overall survival among patients living farther from treatment centers, often mediated by nonadherence and barriers to care [26]. Beyond survival, distance has a measurable negative impact on quality of life. In a cohort of 496 colorectal cancer survivors, greater distance from the treating hospital was associated with significantly worse physical functioning (−4.38 points; 95% CI −8.13 to −0.91) and role functioning (−7.78; 95% CI −12.64 to −2.66), with even greater impairment among women [27]. From a psychosocial perspective, a systematic review including 37 studies demonstrated that patients in rural or remote settings experience higher unmet needs in daily living, increased emotional and practical burden, and significant stress related to travel, family disruption, and work responsibilities [28]. These findings are particularly relevant in pancreatic cancer, where treatment is complex and often requires intensive postoperative care. Long distances may result in reduced family support and limited assistance with essential tasks such as drain management, wound care, and nutritional support—especially in public health systems such as the Brazilian SUS, where access to specialized nursing and structured postoperative care is uneven. Furthermore, prolonged travel and geographic displacement increase psychological stress in an already vulnerable context. Patients frequently return to their home municipalities after surgery. In the event of complications, limited access to the original specialized center may lead patients to seek care in non-specialized hospitals, which may lack experience in managing pancreatic surgery complications. Therefore, even in the absence of a direct impact on survival, travel burden remains highly clinically relevant, as it affects patient experience, continuity of care, and the effective delivery of complex oncologic treatment.

Centralization of pancreatic cancer care is a critical component of improving outcomes, particularly for complex procedures such as pancreaticoduodenectomy. Evidence from the Brazilian public health system demonstrates a clear volume–outcome relationship, with higher-volume centers achieving significantly better short-term outcomes, including lower perioperative morbidity and mortality [29,30]. These findings reinforce the rationale for concentrating complex oncologic surgery in specialized centers with greater technical expertise, multidisciplinary support, and structured perioperative care pathways. However, centralization alone is insufficient and may inadvertently exacerbate disparities if not accompanied by adequate support mechanisms for vulnerable patients. Individuals who must travel long distances for surgery often return to their municipalities without access to specialized postoperative care, including management of surgical drains, wound care, and early recognition of complications. In resource-limited settings, such as many regions within the Brazilian public health system, local facilities may lack the expertise required to manage postoperative pancreatic surgery complications, potentially compromising outcomes despite technically successful procedures. Rather than focusing exclusively on reducing travel distances, efforts should prioritize strengthening the capacity of the health system to support patients referred to high-complexity centers. This includes ensuring structured postoperative follow-up, improving coordination between tertiary referral centers and local healthcare services, and implementing supportive strategies such as transportation assistance and patient navigation programs. An integrated care model that combines centralization of surgical expertise with decentralized support for postoperative management is likely to be essential to balance quality of care with equitable access. In practical terms, this corresponds to a hub-and-spoke model, in which a limited number of high-volume hub centers concentrate complex surgery while a network of spoke facilities closer to patients’ homes delivers diagnosis, pre- and post-operative care, and surveillance under shared protocols. Realizing this model requires specific organizational conditions: (i) formal regionalized referral networks with explicit hub-to-spoke care pathways; (ii) capacity building of local teams, including structured training of physicians and nurses in spoke facilities to recognize and initially manage post-operative complications (e.g., drain and wound care and pancreatic fistula); (iii) telemedicine and tele-mentoring to link local teams with specialists for remote follow-up, second opinions, and early detection of complications—an option that is particularly valuable for patients in remote areas; (iv) patient navigation programs; and (v) transportation and lodging subsidies targeted to socially vulnerable municipalities. SPI-based stratification can be used operationally to decide which municipalities and populations should receive these support measures first.

This study has limitations inherent to its observational design, including the potential for residual confounding and reliance on municipal-level socioeconomic data (SPI), which may not fully capture individual-level vulnerability. Additionally, travel burden was estimated using straight-line distance, which does not account for real-world travel conditions such as transportation infrastructure, travel time, or cost. The database also does not allow detailed assessment of referral patterns, hospital characteristics, treatment sequencing, or patient-level factors such as performance status and social support, all of which may influence both access to care and outcomes. Finally, associations between treatment variables and outcomes should be interpreted cautiously, as treatment selection is inherently influenced by clinical eligibility and survivorship factors. We did not quantify the economic implications of these disparities; reducing avoidable long-distance travel and supporting decentralized post-operative care could plausibly lower both direct patient costs and system-level expenditures (e.g., transport reimbursement, emergency readmissions to non-specialized hospitals, and complication management), but cost variables were unavailable, and formal health-economic evaluation of SPI-targeted interventions remains an important direction for future research. In addition, the registry does not include an explicit rural–urban classification, so the urban-rural dimension of travel burden could only be inferred indirectly from municipal SPI and distance.

## 5. Conclusions

Municipal-level SPI was a strong determinant of healthcare access and the likelihood of receiving surgical treatment for pancreatic cancer. Social and geographic vulnerability directly influence care pathways, revealing structural inequities in access to treatment. SPI-based stratification may serve as a practical tool to identify priority regions for transport support and equitable allocation of oncology services.

## Figures and Tables

**Table 1:** Municipal-level covariates grouped by domain, encompassing geographic, demographic, economic, healthcare, educational, environmental, and equity-related indicators. Variables were obtained from the Brazilian Social Progress Index (Indice de Progresso Social, IPS).

Domain	Variables
**Geography**	Area (km^2^)
**Demographics**	Population
**Economy**	GDP per capita
**Composite Index**	Integrated Social Policy Index (IPS)
**Basic Human Needs**	Nutrition and Basic Medical Care; Water and Sanitation; Housing; Personal Safety
**Foundations of Wellbeing**	Basic Education Access; Information and Communication Access; Health and Wellbeing; Environmental Quality
**Opportunity**	Individual Rights; Personal Freedom and Choice; Social Inclusion; Access to Higher Education
**Healthcare Coverage**	Polio Vaccination Coverage; Avoidable Hospitalizations (PHC); Age-Adjusted Avoidable Mortality (PHC)
**Child and Nutrition Outcomes**	Under-5 Mortality; Undernutrition
**Housing and Infrastructure**	Water Supply via Distribution Network; Adequate Sanitation; Water Supply Index; Water Loss Index; Adequate Waste Collection; Adequate Electricity; Adequate Walls; Adequate Flooring
**Public Safety**	Youth Homicides; Female Homicides; Total Homicides; Transport Accident Mortality
**Education**	Education Dropout (Primary and Secondary); Secondary School Dropout; Secondary School Age-Grade Distortion; IDEB (Primary); Secondary School Failure Rate
**Connectivity**	Mobile Internet Coverage (4G/5G); Fixed Broadband Density; Mobile Phone Density; Mobile Internet Quality
**Lifestyle and Health**	Ultra-Processed Food Consumption; Life Expectancy; Mortality (15–50 years); NCD Mortality; Obesity; Suicides
**Environment**	Urban Green Areas; CO_2_ Emissions per Capita; Heat Spots; Climate Vulnerability Index; Vegetation Suppression
**Justice and Governance**	Access to Human Rights Programs; Minority Rights Actions; Justice Demand Response Index; Social Security Process Response; Family Process Response; Net Case Congestion Rate
**Social Inclusion and Equity**	Access to Culture
**Violence and Discrimination**	Violence Against Indigenous People; Violence Against Women; Violence Against Black People
**Labor Market**	Workers with Higher Education; Employed Women with Higher Education
**Education Quality**	Median High School National Exam Score
**Healthcare Infrastructure**	Number of Hospitals; Number of Physicians; Number of Oncologists; Number of Surgeons; Number of Diagnostic Professionals; Number of Palliative Care Providers

**Table 2:** Baseline characteristics of the cohort.

Characteristic		Value	
**Patients**		***n***	13,478
**Age (years)**		**mean (SD)**	62.3 (±12.9)
**Male**		***n*** **(%)**	6795 (50.4%)
**SPI**		**mean (SD)**	66.4 (±2.9)
**Stage**	**Stage I**	***n*** **(%)**	1018 (7.6%)
	**Stage II**	***n*** **(%)**	1612 (12.0%)
	**Stage III**	***n*** **(%)**	1485 (11.0%)
	**Stage IV**	***n*** **(%)**	6247 (46.3%)
	**Unknown**	***n*** **(%)**	3116 (23.1%)
**Treatment**	**Surgery**	***n*** **(%)**	4448 (33.0%)
	**Chemotherapy**	***n*** **(%)**	6509 (48.3%)
	**Radiotherapy**	***n*** **(%)**	926 (6.9%)
	**Immunotherapy**	***n*** **(%)**	16 (0.1%)
**Travel burden**	**Low (≤10 km)**	***n*** **(%)**	6301 (46.8%)
	**Moderate (10–50 km)**	***n*** **(%)**	2391 (17.7%)
	**High (50–100 km)**	***n*** **(%)**	1497 (11.1%)
	**Extreme (>100 km)**	***n*** **(%)**	3289 (24.4%)

**Table 3:** Unadjusted (crude) and adjusted multivariable models evaluating factors associated with overall survival, stage IV disease at diagnosis, travel distance, and receipt of surgical treatment. For overall survival, hazard ratios (HRs) were estimated using Cox proportional hazards models. For stage IV disease at diagnosis and for surgical treatment, odds ratios (ORs) were estimated from logistic regression models. Travel distance (km) was analyzed as a continuous outcome using linear regression, with coefficients representing the change in distance associated with each variable. Results are presented as effect estimates with 95% confidence intervals (CIs). Age was analyzed as a continuous variable (per year increase), SPI as a continuous variable, and travel burden as a binary indicator (≤10 km vs. >10 km).

Overall Survival				
Variable	Unadjusted HR/OR/Coef. (95% CI)	Unadjusted *p*-Value	Adjusted HR/OR/Coef. (95% CI)	Adjusted *p*-Value
Age (year)	1.024 (1.022–1.025)	<0.001	1.016 (1.015–1.018)	<0.001
Male vs. female	0.852 (0.821–0.884)	<0.001	0.889 (0.853–0.927)	<0.001
Stage IV	2.543 (2.431–2.660)	<0.001	2.228 (2.122–2.340)	<0.001
Surgery	0.406 (0.389–0.423)	<0.001	0.537 (0.511–0.565)	<0.001
Chemotherapy	0.654 (0.630–0.678)	<0.001	0.499 (0.478–0.522)	<0.001
Radiotherapy	0.730 (0.680–0.784)	<0.001	0.960 (0.887–1.038)	0.301
Immunotherapy	0.366 (0.183–0.733)	0.005	0.414 (0.197–0.869)	0.020
SPI (continuous)	0.987 (0.981–0.993)	<0.001	0.994 (0.987–1.002)	0.125
Travel ≤ 10 km	1.014 (0.978–1.052)	0.447		
**Stage IV at diagnosis**				
Age (year)	1.004 (1.001–1.007)	0.017	0.994 (0.990–0.997)	<0.001
Male vs. female	0.831 (0.768–0.899)	<0.001	0.835 (0.767–0.910)	<0.001
Surgery	0.201 (0.184–0.219)	<0.001	0.192 (0.175–0.210)	<0.001
Chemotherapy	0.872 (0.806–0.944)	0.001	0.811 (0.742–0.885)	<0.001
Radiotherapy	0.334 (0.287–0.389)	<0.001	0.339 (0.288–0.400)	<0.001
Immunotherapy	0.790 (0.238–2.744)	0.698		
SPI (continuous)	0.987 (0.974–1.001)	0.068		
Travel ≤ 10 km	0.992 (0.917–1.073)	0.841		
**Travel distance (km)**				
Age (year)	−2.01 (−2.52–−1.49)	<0.001	−1.22 (−1.69–0.76)	<0.001
Male vs. female	−23.6 (−36.9–−10.3)	<0.001	−9.82 (−21.58–1.93)	0.102
Stage IV	0.398 (−14.6–15.4)	0.959		
Surgery	−2.46 (−16.60–11.69)	0.734		
Chemotherapy	−28.14 (−41.44–−14.84)	<0.001	19.83 (7.07–32.59)	0.002
Radiotherapy	1.71 (−24.57–28.00)	0.898		
Immunotherapy	−31.32 (−224.44–161.80)	0.751		
SPI (continuous)	−62.7 (−64.7–−60.7)	<0.001	−62.58 (−64.58–−60.57)	<0.001
**Surgery**				
Age (year)	0.969 (0.966–0.972)	<0.001	0.973 (0.970–0.977)	<0.001
Male vs. female	1.20 (1.12–1.29)	<0.001	1.049 (0.960–1.146)	0.289
Stage IV	0.201 (0.184–0.219)	<0.001	0.192 (0.176–0.210)	<0.001
Chemotherapy	0.836 (0.778–0.898)	<0.001	0.653 (0.596–0.715)	<0.001
Radiotherapy	1.403 (1.223–1.608)	<0.001	1.028 (0.874–1.209)	0.737
Immunotherapy	0.676 (0.189–1.943)	0.499		
SPI (continuous)	1.06 (1.04–1.07)	<0.001	1.040 (1.024–1.056)	<0.001
Travel ≤ 10 km	1.00 (1.00–1.00)	0.734		

**Table 4:** Baseline characteristics, treatment patterns, and travel burden according to quartiles of the Social Progress Index (SPI). Patients were stratified into four groups based on SPI values: Q1 (≤64.7), Q2 (64.7–67.1), Q3 (67.1–68.9), and Q4 (>68.9). Continuous variables are presented as mean (standard deviation), and categorical variables as number (percentage). Group comparisons were performed using analysis of variance (ANOVA) for continuous variables and the chi-square test for categorical variables. Travel burden was evaluated using both continuous distance (kilometers) and predefined categories (low, moderate, high, and extreme). Unknown stage reflects cases with missing or unspecified staging information.

	Characteristic	Q1 (≤64.7)	Q2 (64.7–67.1)	Q3 (67.1–68.9)	Q4 (>68.9)	*p*-Value
**Patients**	**N**	3370	3370	3369	3369	-
**Age**	**years, mean (SD)**	61.5 (12.9)	62.4 (12.8)	62.2 (13.0)	63.2 (12.9)	<0.001
**Male**	***n*** **(%)**	1790 (53.1%)	1728 (51.3%)	1639 (48.6%)	1638 (48.6%)	<0.001
**Stage**	**I, *n* (%)**	227 (6.7%)	231 (6.9%)	314 (9.3%)	246 (7.3%)	<0.001
	**II, *n* (%)**	368 (10.9%)	380 (11.3%)	455 (13.5%)	409 (12.1%)	
	**III, *n* (%)**	344 (10.2%)	347 (10.3%)	444 (13.2%)	350 (10.4%)	
	**IV, *n* (%)**	1500 (44.5%)	1533 (45.5%)	1552 (46.1%)	1662 (49.3%)	
	**Unknown, *n* (%)**	916 (27.2%)	863 (25.6%)	586 (17.4%)	686 (20.4%)	
**Treatment**	**Surgery, *n* (%)**	940 (27.9%)	1049 (31.1%)	1259 (37.4%)	1200 (35.6%)	<0.001
	**Chemotherapy, *n* (%)**	1539 (45.7%)	1595 (47.3%)	1863 (55.3%)	1512 (44.9%)	<0.001
	**Radiotherapy, *n* (%)**	224 (6.6%)	216 (6.4%)	260 (7.7%)	226 (6.7%)	0.151
	**Immunotherapy, *n* (%)**	8 (0.237%)	1 (0.030%)	5 (0.148%)	2 (0.059%)	0.068
**Travel burden**	**Travel distance, km, mean (SD)**	359.4 (684.9)	104.6 (220.7)	43.6 (124.1)	24.8 (124.5)	<0.001
	**Low (≤10 km), *n* (%)**	61 (1.8%)	895 (26.6%)	2284 (67.8%)	3061 (90.9%)	<0.001
	**Moderate (10–50 km), *n* (%)**	837 (24.8%)	1015 (30.1%)	462 (13.7%)	77 (2.3%)	
	**High (50–100 km), *n* (%)**	675 (20.0%)	523 (15.5%)	221 (6.6%)	78 (2.3%)	
	**Extreme (>100 km), *n* (%)**	1797 (53.3%)	937 (27.8%)	402 (11.9%)	153 (4.5%)	

## Data Availability

The corresponding author will provide the datasets used and/or analyzed during the current work upon reasonable request.
